# Two-dimensional Talbot self-imaging via Electromagnetically induced lattice

**DOI:** 10.1038/srep41790

**Published:** 2017-02-06

**Authors:** Feng Wen, Wei Wang, Irfan Ahmed, Hongxing Wang, Yiqi Zhang, Yanpeng Zhang, Abdul Rasheed Mahesar, Min Xiao

**Affiliations:** 1 Key Laboratory for Physical Electronics and Devices of the Ministry of Education & School of Science & Shaanxi Key Lab of Information Photonic Technique, Xi’an Jiaotong University, Xi’an 710049, China; 2 Institute of Wide Bandgap Semiconductors, the School of Electronic and Information Engineering, Xi’an Jiaotong University, Xi’an 710049, China; 3 Department of Electrical Engineering, Sukkur IBA, Sukkur 65200, Pakistan; 4 Department of Physics, University of Arkansas, Fayetteville, Arkansas 72701, USA; 5 National Laboratory of Solid State Microstructures and School of Physics, Nanjing University, Nanjing 210093, China

## Abstract

We propose a lensless optical method for imaging two-dimensional ultra-cold atoms (or molecules) in which the image can be non-locally observed by coincidence recording of entangled photon pairs. In particular, we focus on the transverse and longitudinal resolutions of images under various scanning methods. In addition, the role of the induced nonmaterial lattice on the image contrast is investigated. Our work shows a non-destructive and lensless way to image ultra-cold atoms or molecules that can be further used for two-dimensional atomic super-resolution optical testing and sub-wavelength lithography.

Dimensionality is one of the most defining characteristics of a material; the same chemical compound can exhibit dramatically different properties depending on whether it is arranged in a zero-, one-, or two-dimensional structure. On the atomic scale, two-dimensional materials, such as Bose-Einstein condensates in dilute atomic gases^1^, offer a variety of outstanding properties for fundamental studies and applications^2^. Generally, two optical approaches, on- and off-resonant absorption imaging, are applied to image ultra-cold atoms (or molecules)^3^,^4^. However, on-resonant absorption imaging is limited by its recoil heating and its dynamic range, while off-resonant imaging has the limitation of requiring precisely aligned phase plates or interferometers. Conversely, a good approach is to exploit the Talbot effect^5^,^6^ in which a periodic object’s imaging can be observed at multiples of a certain longitudinal distance without the involvement of optical components if the periodic object is illuminated by incoherent/coherent light^7^,^8^. Due to its potential applications in several new areas of research such as image processing and synthesis, optical testing, photolithography, acousto-optics, electron optics and electron microscopy^9^, this remarkable phenomenon is still being studied and investigated for atomic waves^10^, Bose-Einstein condensates^11^, non-classical light^12^, rogue waves^13^, nonlinear systems^14^,^15^, and pseudo-thermal light ghost imaging^16^ even though it was discovered in 1836.

Recently, with the assistance of Electromagnetically Induced Transparency (EIT) technology^17^,^18^,^19^, periodically modulating the optical response of an ultra-cold atomic (or molecular) medium to a weak probe field, i.e., Electromagnetically Induced Lattice (EIL)^20^,^21^, has been widely studied. By building a spatial-absorption or phase lattice in the atomic sample, a probe beam can be effectively diffracted into high-order diffractions. Such an induced nonmaterial lattice is applicable for realizing optical bistability^22^, probing material optical properties^23^,^24^, and shaping the photonic spectrum^25^,^26^. This technique provides a new non-destructive and lensless choice to image ultra-cold atomic (or molecular) ensembles.

In this paper, we propose another type of non-destructive and lensless imaging system for two-dimensional ultra-cold atomic (or molecular) samples. By exploiting entangled photon pairs as an imaging carrier and generating two-dimensional nonmaterial EIL via two standing-wave fields along the X- and Y-axes, imaging of an ultra-cold atomic (or molecular) sample may be achieved by coincidence measurement of the entangled photon pairs in the detection plane. Further, we show that the image size can be reduced, enlarged, or unchanged simply by adjusting the manner in which the two detectors scan across the imaging beams. In addition, we also show that the optical property of such an induced nonmaterial lattice plays an essential role in image contrast. Our work may broaden the variety of applications used in imaging techniques and prove to be useful for two-dimensional atomic super-resolution optical testing and sub-wavelength lithography as well.

Our imaging system has three advantages. First, it provided lensless imaging and was free of vibrations in the experiment. Second, the transverse resolution of the image can be modulated easily by changing the scanning approach. Third, the imaging visibility can be well controlled by multiple parameters.

## Results

To perform lensless imaging of an ultra-cold atomic (or molecular) ensemble sample, we utilize two perpendicular standing-wave fields to modify the optical response of the medium to the weak probe field. The transmission and dispersion profiles of the weak probe field are manipulated periodically under the condition of an EIT, and then an EIL is established in the sample. Such an optically induced nonmaterial lattice leads to self-imaging of atoms (molecules).

**Figure 1:**
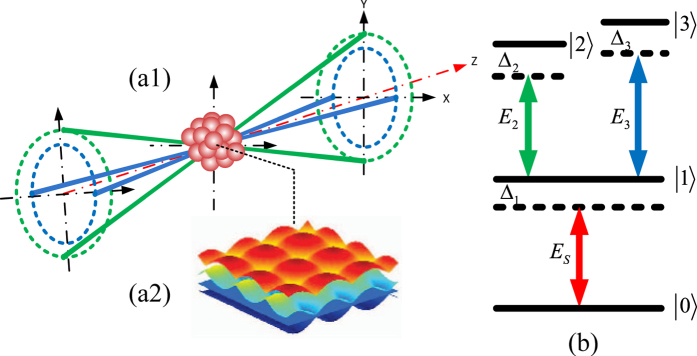
(**a1**) The geometric configuration of the spatial beam with two standing-wave fields and a probe field passing through a cold atomic system, (**a2**) The illustration of an EIL, and (**b**) a closed four-level Y-type atomic system for EISE.

Our scheme includes an entangled photon-pair source, the coupled atomic ensemble and optical field, and the correlation imaging system. In particular, Fig. 1 shows the sample, which consists of an ensemble of closed Y-configuration four-level ultra-cold atoms (or molecules) with length L, while the optical fields are constituted by two standing waves. To produce the optical fields, four fields are injected into the atomic sample symmetrically with respect to z, as shown in Fig. 1(a1), to form two perpendicular standing-wave fields (*E*_2_(x) and *E*_3_(*y*)) in the atomic ensemble (Fig. 1(a2)). In addition, the strong standing wave *E*_2_(x) along the x direction interacts with the atomic ensemble via coupling of the excited upper states |2〉 and meta-stable state |1〉 (|1〉 → |2〉), while the strong standing wave *E*_3_(*y*) along the y direction is coupled to |3〉 and |1〉 (|1〉 → |3〉); see Fig. 1(b). An EIL is generated within the transverse plane of the atomic ensemble (perpendicular to the z-axis). Hence, when a weak signal field *E*_*s*_(x, y) with angular frequency *ω*_*s*_ goes through such a modulated atomic ensemble and couples to the |0〉 → |1〉 transition, the two-dimensional periodic manipulation of the weak signal field is realized. The quantum states of ultra-cold atoms are not influenced during the imaging process as the lattice state is formed from two-dimensional atomic spatial-periodic coherence.

**Figure 2:**
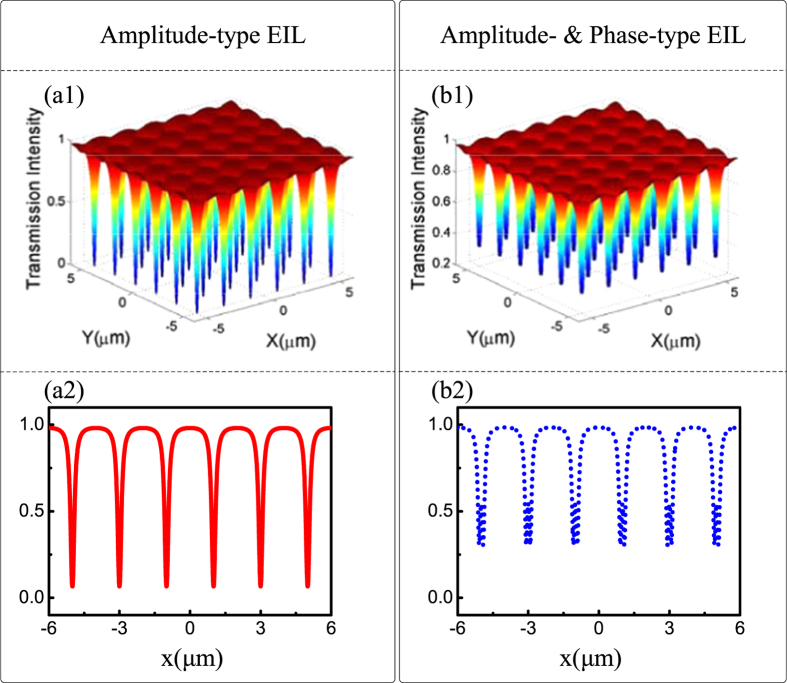
The output profile of the transmitted signal field, (**a1**) An amplitude and (**b1**) A hybrid EIL, as a function of X and Y; (**a2**,**b2**) correspond to the same electromagnetically induced grating.

According to Eq. (8) in the Methods section, the phase modulation about the probe field is absent if *χ*′ = 0, while both phase and amplitude modulation are introduced if 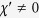 and 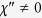. We can see from Eq. (8) that the real parts of *χ* vanish if Δ_1_ = Δ_2_ = Δ_3_ = 0. Therefore, no phase modulation (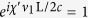) will take place, and the amplitude modulation (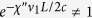) will remain dominant in this case. Figure 2(a1) illustrates the profiles of the probe field at the output surface of the atomic ensemble under Δ_1_ = Δ_2_ = Δ_3_ = 0. Here, the probe beam is significantly absorbed at the transverse locations around the nodes of the standing wave and much less around antinodes. In other words, a phenomenon reminiscent of amplitude-type EIL is realized, where the modulation profile of the probe field is two-dimensional amplitude-intensity dependent.

This interesting phenomenon can be understood from dressed-state theory^27^, where the dressed effect is weaker at locations around nodes and cannot be ignored around the antinodes. The leading probe beam is strongly absorbed around the nodes according to the usual Beer law and is much less absorbed around the antinodes. On the other hand, in the nonresonant case (e.g., Δ_1_ = 0, Δ_2_ = 15, and Δ_3_ = 15), both phase modulation (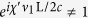) and amplitude modulation (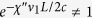) are introduced to modulate the probe field. To illustrate this more clearly, Fig. 2(a2,b2) display the corresponding modulation in one dimension only. As shown in Fig. 2(b2), a spatial hybrid EIL (both amplitude and phase modulation) is formed. In contrast to Fig. 2(a2), the probe field experiences a rapid phase change at the nodes. On the other hand, due to the introduction of Electromagnetically Induced Absorption (EIA) at the antinodes (see Fig. 2(b2), the peaks in the EIT window), the intensity of the probe field is significantly decreased in contrast with amplitude-type EIL.

## Discussion

**Figure 3:**
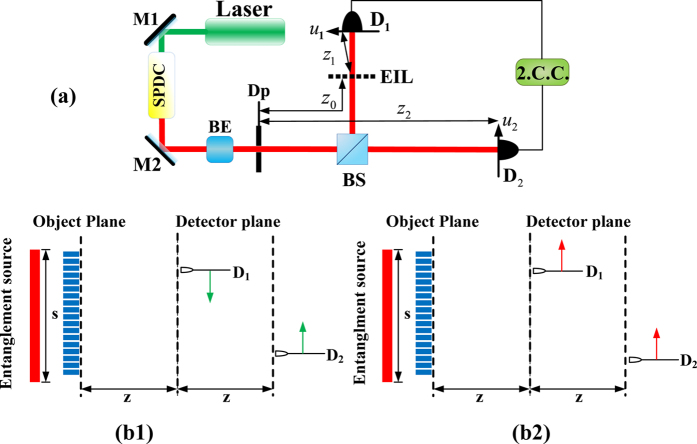
Setup to realize the imaging of 2D ultra-cold atomic (or molecular) ensembles. EIL: electromagnetically induced lattice; BE: beam expander; Dp: diaphragm; M1, M2: mirrors; and BS: beam splitter. Two photon detectors moved together with opposite directions and the same direction in (**b1**,**b2**), respectively.

Now, we study the self-imaging in a typical quantum-imaging configuration (see Fig. 3) in which a pair of entangled photons (signal and idler photons) is generated through spontaneous parametric down-conversion (SPDC) in BBO crystal cut for type-I phase matching and then are separated by a beam splitter (BS).

The distances from the outer surface of the crystal to the atomic ensemble, *D*_2_ and *D*_1_, are *z*_0_, *z*_2_, and *z*_1_, respectively. In the signal arm, the atomic ensemble is inserted between the BS and the bucket detector *D*_1_, where it is coupled with signal photons via the atomic-transition channel |0〉 → |1〉. As discussed in Sec. III, it’s the optical-transfer function, which is characterized by 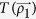. In the idler arm, the idler photon is employed as a trigger and detected by the reference detector *D*_2_. The signal and idler photons are transmitted along the signal arm and idler arm, respectively and are subsequently measured by two photon detectors.

### Theoretical Analysis

Some interesting conclusions can be directly drawn from Eq. (15) in the Methods section. First, the first exponential term (the “localization” term) in Eq. (15) describes the phase change of the diffraction orders along the propagation directions, and indicates whether self-imaging occurs or not. That is, the transmitted-object light amplitudes are repeated only if self-imaging occurs in plane, where all diffraction orders are in phase and interfere constructively. Second, the effective diffraction length corresponding to EIL Self-imaging is equal to *Z*_*eff*_ = *z*_1_(*z*_0_ + *z*_2_)/(*z*_1_ + *z*_0_ + *z*_2_). Third, it is apparent that the self-imaging occurs at *Z*_*eff*_ = *mz*_*T*_/2, where *z*_*T*_ = (*a*^2^ + *b*^2^)/2*λ*_*s*_ is the Talbot length, and *m* is a positive integer, referred to as the *m*-th self-imaging plane. Specifically, if *m* is an odd integer, the self-image is shifted by a half-period with respect to that obtained when *m* is an even integer.

Different from traditional self-imaging, generally, the measurement of two-photon EIL Self-imaging involves the combinational adjustment of both detectors’ (*D*_1_ and *D*_2_) positions. From Eq. (16) in the Methods section, the magnification of the atomic ensemble closely depends on 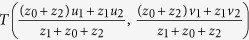, while 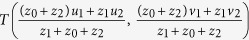 is determined by the scanning approach of both detectors across the signal and idler beams.

We focus on the following three special scanning approaches. In the first, both detectors (*D*_1_ and *D*_2_) are scanned synchronously across the signal and idler beams with identical directions, i.e., transverse constraints *u*_1_ = *u*_2_ and *v*_1_ = *v*_2_ are satisfied. Therefore, when *D*_2_ is scanned along the longitudinal z direction, Eq. (16) is reduced to 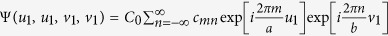, and the size of the image is exactly the same as that of the original EIL or of the traditional self-image (the corresponding magnification factor is *M*_1_ = 1). In the second scanning approach, one of the detectors (*D*_1_ or *D*_2_) is fixed at its origin while the other is moved along the X- and Y-axes. For example, if *D*_2_ is scanned, and *D*_1_ is kept at the origin (*u*_1_ = 0, *v*_1_ = 0), the biphoton amplitude in Eq. (16) is of the form 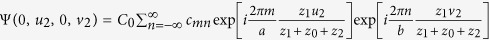. Compared with the size of the original EIL, the self-image is magnified by a factor *M*_2_ = 1 + (*z*_0_ + *z*_2_)/*z*_1_. However, if *D*_1_ is scanned and *D*_2_ is fixed at the origin (*u*_2_ = 0, *v*_2_ = 0), Eq. (16) is reduced to 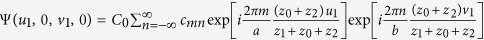, and the self-image is magnified by *M*_3_ = 1 + *z*_1_/(*z*_0_ + *z*_2_). In the third scanning approach, both detectors are scanned synchronously across twins beams but in opposite directions, i.e., *u*_1_ = −*u*_2_ and *v*_1_ = −*v*_2_. The two-photon amplitude is 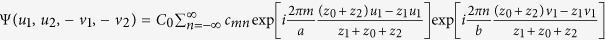, and the corresponding magnification is *M*_4_ = 1 + 2*z*_1_/(*z*_0_ + *z*_2_ − *z*_1_). Therefore, in contrast with traditional self-imaging, the Talbot carpet pattern can be arbitrarily modulated in the second and third scanning approaches.

### Numerical examples and discussion

In the previous section, based on Eq. (15) in the Methods section, some features of two-photon EIL Self-imaging were theoretically predicted and analysed. In this section, we test these predictions using numerical simulation. For convenience, we assume that the generated entangled photon pairs have the same wavelength *λ*_*s*_ = *λ*_*i*_ = 883.2 nm and that the periods of the EIL along the X and Y components are equal: *a* = *b* = 2 μm.

**Figure 4:**
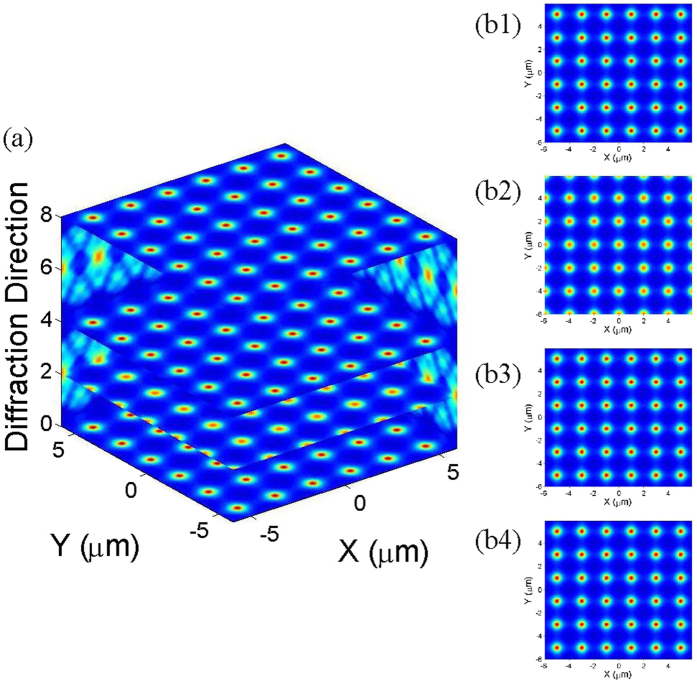
(**a**) Self-imaging carpets of a two-dimensional ultra-cold atomic ensemble versus x, y and z, obtained by scanning *D*_1_ and *D*_2_ using the first scanning approach. The four panels are the contour plots of the Self-image at (**b1**) 0, (**b2**) *z*_*T*_/4, (**b3**) *z*_*T*_/2, and (**b4**) *z*_*T*_. The parameters are Δ_1_ = Δ_2_ = Δ_3_ = 0, and *G*_2_ = *G*_3_ = 15 MHz.

As analysed above, the two-photon EIL Self-image is determined not only by the scanning approach of the two detectors across the signal and idler beams but also by the interaction circumstances of the atomic ensemble and the light field. We first focus on resonant atom-light interaction circumstances, i.e., Δ_1_ = Δ_2_ = Δ_3_ = 0, under the first scanning approach. Hence, the coupled atomic-field ensemble in this case is of amplitude-type EIL; see Fig. 2(a). In Fig. 4, the main results of second-order self-imaging are presented, where *D*_1_ and *D*_2_ are scanned synchronously across the signal and idler beams along the X- and Y-axes in identical directions. During this process, the distance between the atomic ensemble and *D*_1_ is fixed (*z*_1_ = *z*_*T*_), and the distance to the crystal is *z*_0_ = *z*_*T*_. We can see from Fig. 4(a) that a typical 2-dimensional Talbot carpet pattern is produced, while the transverse and longitudinal resolutions of the diffraction patterns are unchanged when *D*_2_ is scanned along the longitudinal z direction. To obtain a more intuitive display, in Fig. 4(b1–b4), we obtain the 2-dimensional diffraction patterns at the positions *z*_2_ = 0, *z*_*T*_/2, *z*_*T*_ and 2*z*_*T*_, respectively. Specifically, when *D*_2_ is fixed at *z*_2_ = 0, the image-revival size is same as the original EIL size of 2 μm; see Fig. 4(b1). Furthermore, the transverse resolutions of the Talbot carpet patterns are still unchanged if D_2_ is moved to *z*_*T*_/2, *z*_*T*_ and 2*z*_*T*_; see Fig. 4(b2–b4). The only difference between Fig. 4(b3,b1) is that the image obtained at *z*_2_ = *z*_*T*_/2 is shifted by a half period relative to the image obtained at *z*_2_ = *z*_*T*_/2. These results fit very well with the predictions described in the Theoretical Analysis section.

This interesting phenomenon can be understood based on Eq. (16) in the Methods section, where the expression of 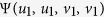 corresponding to the first scanning approach is independent of the positions of the two detectors. In other words, the amplification factors *M*_1_ at *z*_2_ = *z*_*T*_/2, *z*_*T*_ and 2*z*_*T*_ are equal to one. Therefore, the sizes of the diffraction patterns are all equal to that of the original EIL, and the transverse resolution is also fixed. We also indicated that this interesting phenomenon still holds even if the detectors are not at the same distance from the light source.

**Figure 5:**
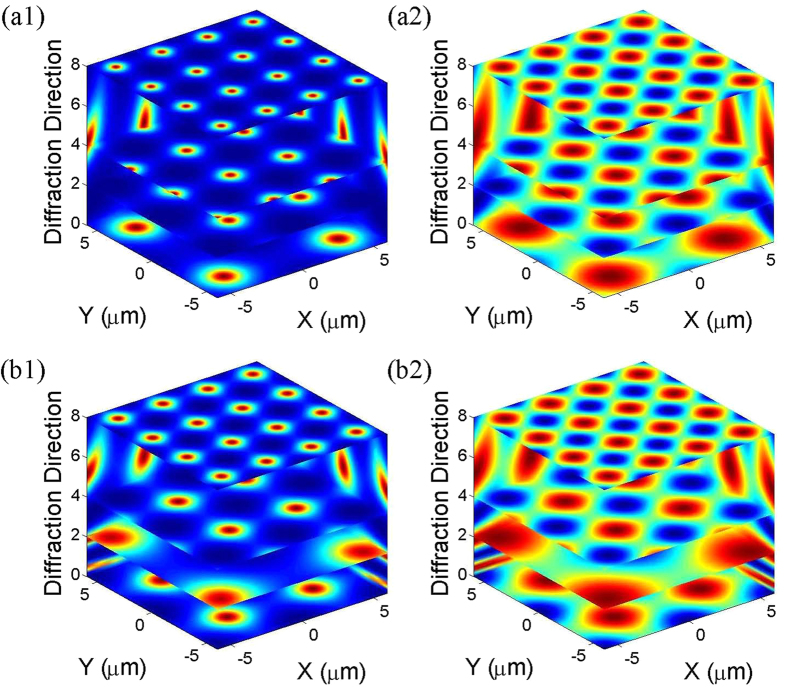
(**a**,**b**) Self-imaging carpets of a two-dimensional ultra-cold atomic ensemble versus x, y and z, obtained by scanning *D*_1_ and *D*_2_ using the second and third scanning approaches, respectively; (**a1**,**b1**) are the self-images of amplitude EIL (Δ_1_ = Δ_2_ = Δ_3_ = 0), while (**a2**,**b2**) correspond to hybrid EIL (Δ_1_ = 0, Δ_2_ = 15, and Δ_3_ = 15). The other parameters are *G*_2_ = *G*_3_ = 15 MHz.

We also investigate the evolution of Talbot diffraction patterns by scanning the two detectors using the second and third scanning approaches, setting Δ_1_ = Δ_2_ = Δ_3_ = 0 as before; see Fig. 5(a1,b1), respectively. Some interesting features are observed in these two cases. As shown in Fig. 5(a1), by setting *z*_1_ = 3*z*_*T*_/2 and *z*_0_ = *z*_*T*_/2, typical Talbot carpet patterns are produced, and the 2-dimensional diffraction patterns are gradually reduced when *D*_2_ is scanned along the longitudinal direction. For instance, at *z*_2_ = 0, the image is revived at four times the size of the original EIL image, and at *z*_2_ = *z*_*T*_/2, the image is shifted by a half period along both the X and Y directions with respect to the pattern at *z*_2_ = 0, with a period of 5 μm. Furthermore, at *z*_2_ = *z*_*T*_, the diffraction pattern is repeated with a period of 4 μm. If *D*_2_ is further moved along the longitudinal direction by *z*_*T*_, i.e., *z*_2_ = 2*z*_*T*_, compared with the imaging at *z*_2_ = 0, it can be seen that the imaging spots are 3.2 μm. This can be understood based on the effective diffraction lengths corresponding to *z*_2_ = 0, *z*_*T*_/2, *z*_*T*_ and 2*z*_*T*_, which are 3*z*_*T*_/8, 3*z*_*T*_/5, 3*z*_*T*_/4, and 15*z*_*T*_/16, respectively, leading to amplification-factor enlargement with *M*_3_ = 4, 2.5, 2, and 1.6.

In the third scanning approach, setting *z*_1_ = *z*_*T*_/2 and *z*_0_ = *z*_*T*_/4, the transverse resolutions of the Talbot carpet patterns are first decreased and then increased when *D*_2_ is moved along the longitudinal direction; see Fig. 5(b1), which is different from Fig. 5(a1). Specifically, the diffraction patterns of EIL change from 6 μm, 10 μm, and 6.6 μm, to 3.14 μm if *D*_2_ is moved to *z*_2_ = 0, *z*_*T*_/2, *z*_*T*_ and 2*z*_*T*_, respectively, and the corresponding magnification factors are 3, 5, 3.33, and 1.57. As indicated in the Theoretical Analysis section, 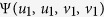 is very sensitive to changes in the positions of the two detectors, and the diffraction patterns are magnified by 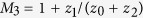 (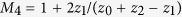) in the second (third) scanning approach. Therefore, when *D*_2_ is moved along the longitudinal direction, the transverse diffraction pattern is gradually decreased in the second scanning approach, which is further increased and then decreased in the third scanning approach.

As indicated by Figs 2 and 5, the visibility of the self-image is determined by the interactions between the laser fields and the ultra-cold atomic (or molecular) ensemble, i.e., amplitude-type EIL or hybrid-type EIL. That is, the visibility of imaging will be increased if the gap between the nodes and antinodes of the standing wave is expanded. On the other hand, considering the second-order spatial-correlation function *G*^(2)^(*u*_*m*_, *u*_*n*_), we found that the spatial resolution closely depends on the spatial-correlation term *S*in*c* (Δ*θ*(*u*_*m*_ + *u*_*n*_)/*λ*)^16^; here, Δ*θ* = 2*πr*/*z* is the angular size of the source with respect to the detector plane, and *λ* is the wavelength of the imaging light. In other words, the larger the coefficient of Δ*θ*/*λ*, the narrower of full width at half maximum (FWHM), leading to higher spatial resolution. Therefore, the spatial-correlation term is reduced to 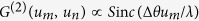 if we move two photon detectors together with opposite directions during scanning; see Fig. 3(b1). In contrast, as shown in Fig. 3(b2), if we move two photon detectors together with the same direction during scanning, the FWHM of the spatial-correlation peak is reduced to 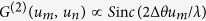, and the spatial resolution can be significantly improved in this scanning approach.

The Talbot carpet patterns shown in Fig. 5(a2,b2) have the same conditions as those in Fig. 5(a1,b1), except Δ_1_ = 0, Δ_2_ = 15, and Δ_3_ = 15 (hybrid-type EIL). From the comparison between Fig. 5(a1,a2) (or Fig. 5(b1,b2)), it is apparent that the longitudinal (transverse) resolutions of the images and the location of the Talbot plane in the hybrid-type EIL case exactly coincide with those of the amplitude-type EIL case. All of these properties are independent of the introduced phase modulation. Due to the introduction of phase modulation, however, we noticed that images under resonant conditions (amplitude-type EIL) are clearer than hybrid-type EIL (off-resonant) images, and the maximum amplitude contrast is decreased in hybrid-type EIL images. All of these results agree well with the predictions drawn from Eq. (16) in the Methods section.

Before proceeding to the next section, some points need to be emphasized. Indeed, compared with the traditional imaging approach, our method has some defects. However, these defects may be solved by introducing new theories and methods. (a) Considering that two photon detectors are needed for simultaneous two-dimensional scanning, leading to an extremely long measurement time, we use Charge-Coupled Devices (CCD) instead of two photon detectors because the imaging results of an EIL are directly present on CCDs in two dimensions, and we also use an ultrafast photon detector to shorten the detection time. (b) Because we are missing the colour information of the object, we can adopt a multi-wavelength ghost-imaging method to realize multi-colour imaging. (c) To improve the image quality (in terms of resolution and contrast), we can utilize a higher-order correlation-imaging method.

Conventional self-imaging research has been limited to use real lattices for imaging. In our scheme, the lattice state is the periodic intensity pattern on the output surface of the atomic (or molecular) ensemble, and such nonmaterial EIL can be effectively modulated via EIL. This difference distinguishes our scheme from the conventional self-imaging research. Compared with the conventional Talbot imaging, such EIL self-imaging does not require any converging optical elements, i.e., imaging lens, which greatly simplifies the experimental setup. Another advantage of the newly proposed EIL self-imaging system is that the spatial period can be adjusted easily by varying the angle between *E*_2_ and 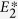 (*E*_3_ and 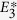), while the spatial period in conventional Talbot imaging is fixed. Thus, we provide a better (optical) way to observe an ensemble of various atoms (or molecules) without changing the imaging system. More importantly, one major disadvantage of the conventional Talbot imaging is that the transverse resolution is limited to the wavelength of the probe field. In our scheme, however, the optimized transverse resolution of the image can be achieved by selecting the scanning approaches of both detectors across the imaging beams. This effect can also be further applied with pseudothermal light source to achieve sub-Rayleigh images. Generally, the EIL self-imaging method presented here not only enriches conventional imaging techniques but also offers a new method for imaging in a broad range of applications.

## Conclusion

In summary, assisted by an EIL, we propose a theoretical scheme to image two-dimensional cold atoms where Bose-Einstein condensation (BEC) on a chip (or optical lattice) can be non-locally observed by coincidence measurement. By changing the scanning approaches of both detectors across the imaging beams, we show that the transverse resolution of the image can be modulated, i.e., reduced, enlarged, or unchanged. We also indicate that the optical properties of induced nonmaterial EIL play an essential role in image contrast. Further, the development of our proposed method will be presented in two-dimensional atom super-resolution optical testing and sub-wavelength lithography and could be a useful tool for quantum-information science as well.

## Methods

### Evolution of the Probe Field in an Atomic Ensemble

In the interaction picture, the effective Hamiltonian under the electric-dipole approximation and the rotating-wave approximation is expressed as (*ħ* = 1)


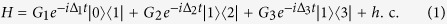


where *G*_*i*_ = *μ*_*ij*_*E*_*i*_/*ħ* are the Rabi frequencies of the optical pumping field, and the laser-field detunings from the transitions |0〉 → |1〉, |1〉 → |2〉 and |1〉 → |3〉 are defined as Δ_1_ = *ω*_1_ − *ω*_10_, Δ_2_ = *ω*_2_ − *ω*_21_ and Δ_3_ = *ω*_3_ − *ω*_31_, respectively, with *ω*_*ij*_ = *ω*_*i*_ − *ω*_*j*_, (*i, j* = 0, 1, 2, 3).

By using the Liouville equation, the coupled system equations are obtained:


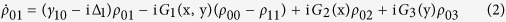



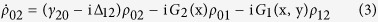



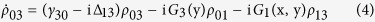



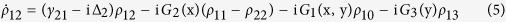



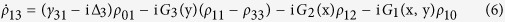



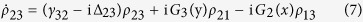


where 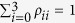, 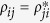 and Δ_12_ = Δ_1_ + Δ_2_, Δ_13_ = Δ_1_ + Δ_3_ and Δ_23_ = Δ_2_ − Δ_3_. *γ*_32_, *γ*_30_, and *γ*_20_ are decoherence rates, and *γ*_31_, *γ*_21_, and *γ*_10_ are decay rates of upper levels.

By solving Eqs (2–7), with the assumption that the atomic ensemble is initially in its ground state |0〉 (i.e., *ρ*_00_(0) = 1), the linear susceptibility of the coupled system at *ω*_*s*_ is obtained:


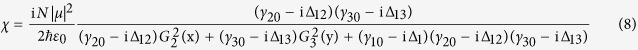


where *μ* is the atomic dipole moment, *N* is the atomic density, and *ε*_0_ is the vacuum permittivity. 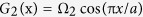 (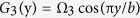) is the Rabi frequency of the strong standing wave along the X (Y) direction where Ω_2_ and Ω_3_ are the amplitudes of the two laser fields and are assumed to be real for simplicity. *a* (*b*) is the corresponding spatial period, as shown in Fig. 1(a), and can be made arbitrarily smaller or larger than the wavelength of probe field *E*_1_ by varying the angle between the two wave vectors of *E*_2_ and 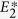 (*E*_3_ and 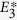).

To describe the interaction between the probe fields and the two-dimensional electromagnetically modulated cold-atom ensemble, the effective Hamiltonian operator 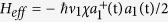 is introduced, and the propagation dynamics of the probe field within the atomic ensemble is obtained as 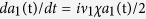. Using *t* = *L*/*c*, the transmission profile of the probe field at the output surface of the atomic ensemble is 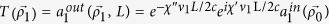; here, the susceptibility (*χ* = *χ*′ + *iχ*″) in Eq. (8) is written in terms of its imaginary *χ*″ and real *χ*′ parts to describe the amplitude change and phase shift, respectively, and the input profile of the probe field 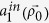 is assumed to be a plane wave (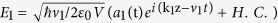).

### Second-order EIL Self-imaging

Using Glauber’s quantum-measurement theory, the second-order coincidence-counting rate for the two-photon self-imaging process is expressed as^26^


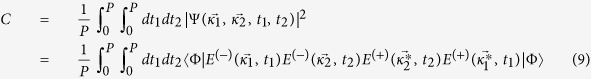


where 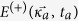 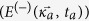 is the positive (negative) frequency part of 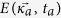 (*a* = 1, 2). 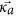 and *t*_*a*_ are the transverse coordinate and triggering time, respectively, in the *a*–th detection plane. *P* is chosen to capture the coincidence count, and |Φ〉 is the biphoton state at the output surface of the nonlinear crystal. According to perturbation theory^28^,^29^, |Φ〉 can be written as 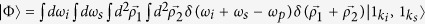, where *ω*_*m*_, 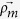 and *k*_*m*_ (*m* = *s, i*) are the angular frequency, transverse coordinate, and wave vectors of the entangled photon, respectively. The perfect frequency (*δ*(*ω*_*i*_ + *ω*_*s*_ − *ω*_*p*_)) and spatial phase matching 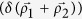 indicate that the biphoton generated from spontaneous parametric down-conversion (SPDC) is entangled in both the frequency and spatial domains.

Then, by taking the propagation effect into account, the optical field at the detector is transferred:


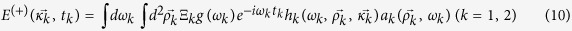


where *g*(*ω*_*k*_) is the narrow bandwidth of the filter function peaked with central frequency Ω_*k*_ (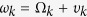 and 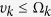), and 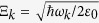. 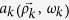 is the photon-annihilation operator, which satisfies 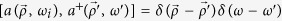. In Eq. (10), Green’s function 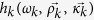 describes the propagation mode *ω*_*k*_ from the output surface of the crystal 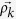 to the detector with transverse point 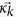. Assuming the paraxial approximation, which always holds, the impulse-response functions for the signal arm 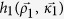 and idler arm 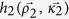 will take the following forms.


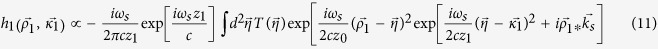



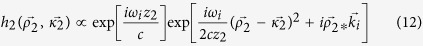


where the transmission function is described as 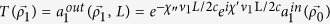. By substituting Eqs (10–12) into the biphoton amplitude 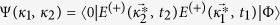, and completing the integration on transverse mode 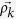, the two-photon amplitude is obtained:


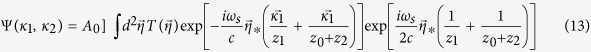


where the irrelevant terms have been absorbed into *A*_0_, and 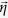, 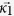 and 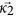 are the transverse coordinates at the output surface of the atomic ensemble, detected at planes *D*_1_ and *D*_2_, respectively.

In fact, 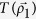 can be expanded into a 2-dimensional Fourier series as


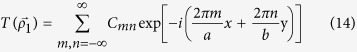


where *a* and *b* are the spatial periods along the X and Y directions, and *C*_*mn*_ is the 2-dimensional Fourier coefficient. The biphoton amplitude can be simplified by substituting Eq. (14) into Eq. (13) and completing the integration on 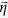:


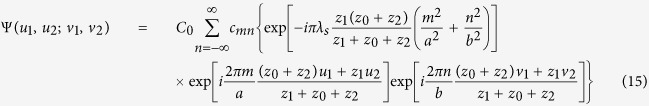


where [*u*_*k*_, *v*_*k*_] (k = 1, 2) are coordinates along directions [X, Y], respectively, in detection plane *D*_*k*_.

The “localization” terms are set to 1 in self-image planes, so Eq. (15) can be further reduced:


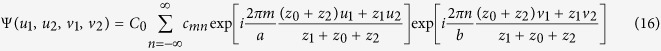


Based on Eqs (15) and (16), we discuss and analyse many interesting properties of self-imaging in the Discussion section.

## Additional Information

**How to cite this article**: Wen, F. *et al*. Two-dimensional Talbot self-imaging via Electromagnetically induced lattice. *Sci. Rep.* **7**, 41790; doi: 10.1038/srep41790 (2017).

**Publisher's note:** Springer Nature remains neutral with regard to jurisdictional claims in published maps and institutional affiliations.
